# Nutrient Intake Is Associated with Longevity Characterization by Metabolites and Element Profiles of Healthy Centenarians

**DOI:** 10.3390/nu8090564

**Published:** 2016-09-19

**Authors:** Da Cai, Shancang Zhao, Danlei Li, Fang Chang, Xiangxu Tian, Guohong Huang, Zhenjun Zhu, Dong Liu, Xiaowei Dou, Shubo Li, Mouming Zhao, Quanyang Li

**Affiliations:** 1 College of Light Industry and Food Engineering, Guangxi University, Nanning 530004, China; caida0906@163.com (D.C.); HGH00809@163.com (G.H.); zhenjunz07@163.com (Z.Z.); dongliu1207@sina.com (D.L.); lsb1696@163.com (S.L.); 2 Institute of Agricultural Quality Standards and Testing Technology Research, Shandong Academy of Agricultural Sciences, Jinan 250100, China; shancangzhao@126.com; 3 College of Life Sciences, Tianjin Normal University, Tianjin 300387, China; danleili@163.com; 4 Institute of Agro-Products Quality Testing, Jinan Academy of Agricultural Sciences, Jinan 250316, China; changfang6@sina.com (F.C.); xiangxut@yeah.net (X.T.); 5 Food and Biological Technology Department, Guangxi Polytechnic College, Nanning 530226, China; 6 Veterans Affairs Boston Healthcare System, Department of Neurology, Harvard Medical School, West Roxbury, MA 02132, USA; Xiaowei_Dou@hms.harvard.edu

**Keywords:** centenarian, nutrient intake, metabolite, element, longevous region, pattern recognition, partial least squares-discriminant analysis

## Abstract

The relationships between diet and metabolites as well as element profiles in healthy centenarians are important but remain inconclusive. Therefore, to test the interesting hypothesis that there would be distinctive features of metabolites and element profiles in healthy centenarians, and that these would be associated with nutrient intake; the short chain fatty acids (SCFAs), total bile acids and ammonia in feces, phenol, *p*-cresol, uric acid, urea, creatinine and ammonia in urine, and element profiles in fingernails were determined in 90 healthy elderly people, including centenarians from Bama county (China)—a famous longevous region—and elderly people aged 80–99 from the longevous region and a non-longevous region. The partial least squares-discriminant analysis was used for pattern recognition. As a result, the centenarians showed a distinct metabolic pattern. Seven characteristic components closely related to the centenarians were identified, including acetic acid, total SCFA, Mn, Co, propionic acid, butyric acid and valeric acid. Their concentrations were significantly higher in the centenarians group (*p* < 0.05). Additionally, the dietary fiber intake was positively associated with butyric acid contents in feces (*r* = 0.896, *p* < 0.01), and negatively associated with phenol in urine (*r* = −0.326, *p* < 0.01). The results suggest that the specific metabolic pattern of centenarians may have an important and positive influence on the formation of the longevity phenomenon. Elevated dietary fiber intake should be a path toward health and longevity.

## 1. Introduction

Health and longevity are the eternal pursuit of human beings. Diet is an important factor affecting health and longevity [1,2]. Appropriate dietary alterations, such as dietary restriction, can promote health and increase the life span of laboratory model organisms [3], but malnutrition, nutritional excess or imbalance accelerates the aging process and causes a variety of geriatric diseases in humans [4]. Besides, dietary compositions also influence the metabolism of an individual and contribute to a variation in metabolites.

Metabolite levels are important indicators of human health and longevity. Many diseases and inflammations are associated with metabolic imbalances [5]. Studies show that short chain fatty acids (SCFAs) (such as butyric acid) reduce the risk of gastrointestinal diseases and cancer [6], and also exert important immunomodulatory roles [7]. In addition, certain essential elements, such as Mn, Se and Zn, are critical to the human metabolism and aging [8,9]. Lv et al. reported that the higher Mn levels in the hair of centenarians were conducive to their long lifespan [10]. Besides, Se is associated with the prevention of some diseases such as cancer and Alzheimer's disease, and also has important impacts on longevity [11]. As for elderly people, appropriate levels of metabolites and elements in elderly people play important roles in the prevention of geriatric diseases and the maintenance of normal immune functions [12]. Therefore, monitoring the contents of relevant metabolites and elements is of great significance for human health and longevity. As centenarians represent the model of healthy and successful aging well [13], it is necessary to explore the features of metabolites and element profiles in healthy centenarians. Nevertheless, the relevant studies on the metabolites and element profiles in healthy centenarians are still limited. Furthermore, previous studies also have not specifically assessed the correlation between metabolites and nutrient intake of healthy elderly people over 80 years of age.

It is noteworthy that human health and longevity are associated with overall metabolites and element levels rather than individual ones. To comprehensively analyze the overall influence of various indexes on health and longevity, it is necessary to use a multivariate statistical analysis method to perform pattern recognition. Partial least squares-discriminant analysis (PLS-DA) can be applied to construct the classification model and identify characteristic components. More importantly, PLS-DA has advantages in solving the classification problem of a very small number of samples [14]. Thus, considering the limited sample size of this study, PLS-DA is used to analyze the comprehensive impact of metabolite and element levels on health and longevity. So far, the studies using PLS-DA for pattern recognition mainly focus on investigating the relationship between specific diseases and levels of metabolites or elements in the human body [15,16]. However, no work has been reported to use PLS-DA for comprehensive evaluation of the characteristics of metabolites and elements in healthy centenarians.

There is a marvelous phenomenon in Bama county, Guangxi province, China, where the ratio of centenarians was the highest in China. According to the Population Census of China in 2010 [17], there were 80 centenarians in the population of 224,637, reaching to a ratio of 35.6 centenarians per 1 × 10^5^ persons [18]. The ratio of centenarians in this region far exceeds the world longevity county standard specified by the United Nations (7.5/100,000) [19]. Nevertheless, few researches have been focused on the metabolites and element profiles of elderly people living in Bama longevous region (LR) because of their traditional and conservative lifestyle.

In this study, based on the rare phenomenon of healthy aging and extreme longevity, the hypothesis that there would be remarkable and distinctive features of metabolites and element profiles in healthy centenarians from Bama LR, and that these would be associated with nutrient intake, should be of interest. To test this hypothesis, we enrolled 49 healthy centenarians in the LR (LRC group), 59 healthy elderly people aged 80–99 in the LR (LRE group) and 61 healthy elderly people aged 80–99 from a non-longevous region (NLR) (NLRE group). The levels of metabolites and elements in the three groups were analyzed. PLS-DA was used to further explore the overall impact of the metabolites and element levels on health and longevity, and to identify the characteristic components closely related to the centenarians. Moreover, we assessed the relationships between metabolites or element concentrations and dietary nutrient intake. This will be very important for a better understanding of the relationship between diet and longevity from the perspective of metabolism, which will provide a theoretical basis for a healthy diet.

## 2. Materials and Methods

### 2.1. Participants

The study was conducted in the Bama district and Xixiangtang district of the Guangxi province, China, during the period from 2013 to 2015. The Xixiangtang district was selected as the non-longevous region (NLR), where the ratio of centenarians was relatively lower [18] (only three centenarians in the population of 1,156,173 [17], a ratio of 0.26 centenarians per 1 × 10^5^ persons), and the climatic environment was similar with the Bama district. By means of a distribution of flyers and information sessions in the residential community, and thorough community screenings according to the population census data, we enrolled healthy centenarians in the LR (LRC group), and elderly people aged 80–99 in the LR (LRE group) and the NLR (NLRE group). The age of participants was validated by the following steps. First, the names, dates of birth, and addresses of participants were obtained by the population census data. Second, the participants were required to provide a valid identification card or household register booklet, which lists the name, date of birth, and other important demographic information. Third, the Twelve Animal Year of birth according to the traditional Chinese calendar should match the official year of birth [20]. Fourth, the age of participants was also checked by using the generation of family and the familial reconstitution method. Finally, neighbors of participants and villagers were also consulted to confirm the age of participants. To guarantee that each participant was a relatively healthy elderly person, the following screening criteria were adopted. Ineligibility criteria included uncontrolled hypertension, unstable type 1 diabetes, major cardiovascular diseases, any form of cancer, severe dementia or dysphasia, hepatitis, renal impairment, endocrine disease, inflammatory diseases, compromised immune status (AIDS, multiple myeloma, chronic lymphatic leukaemias), a physical disability that limits walking or dependency on others for activities of daily living, cognitive impairment, gastrointestinal disorders and/or food allergies. In addition, no antibiotics or drugs known to affect experimental endpoints, or nutritional supplements were taken during the course of this study. During recruitment and screening, each participant was required to complete a questionnaire on their medical history to provide health information.

**Figure 1:**
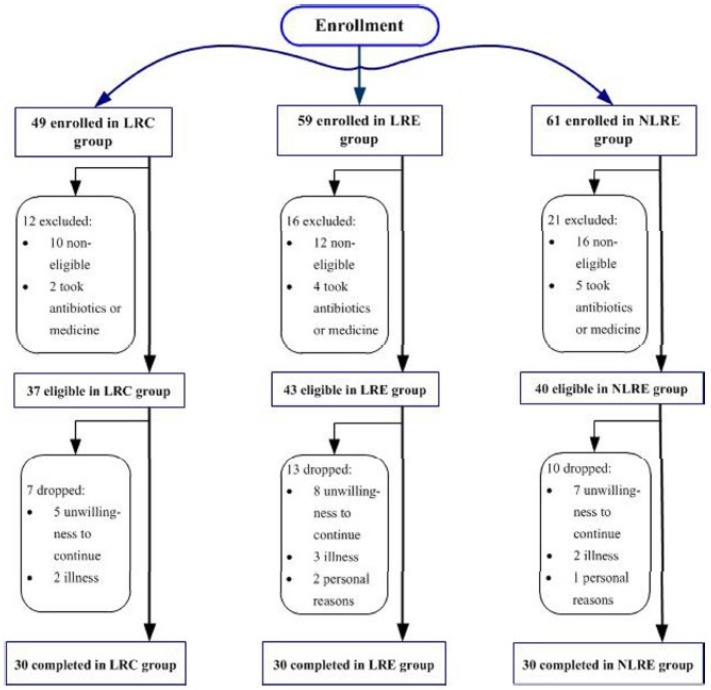
Flow diagram of participants during the study.

Forty-nine centenarians were enrolled in the LRC group, and 59 elderly people from the LR and 61 elderly people from the NLR were enrolled in the LRE group and NLRE group, respectively. Among these volunteers, 37 centenarians in the LRC group, 43 elderly people in the LRE group and 40 elderly people in the NLRE group met the screening criteria and started the study. Of these, 30 centenarians in the LRC group (11 male/19 female, age: 103 ± 3 years), 30 elderly people in the LRE group (12 male/18 female, age: 87 ± 5 years), and 30 elderly people in the NLRE group (13 male/17 female, age: 88 ± 4 years) completed the study protocol (Figure 1). The characteristics of the participants are shown in Table 1. There is no significant difference in age between the LRE group and NLRE group (*p* > 0.05), as well as in sex ratio among the three groups (*p* > 0.05).

All study procedures were reviewed and approved by the Institutional Ethics Review Board of Guangxi University prior to initiation of this study. Written informed consent was obtained from each participant.

### 2.2. Assessment of Dietary Nutrition Status

The assessment of dietary nutrition status was carried out by means of four season consecutive 7-day weighed dietary records (28-day WDRs) [21], which were conducted in January, April, July, and October. During the dietary survey, all of the participants were required to adopt the mode of individual dining, and were advised to maintain usual eating habits. All food and drinks consumed by the participants were weighed and recorded by the trained investigators using electronic food scales, measuring cups, and spoons. For unmeasurable food, standard portion size was used. For the purposes of quality control, ten percent of the dietary records were reviewed and checked by the trained dietitians on a dietary survey site. The dietary data was transformed to dietary nutrient intake based on the Chinese food composition tables [22]. Average daily nutrient intake was calculated by multiplying the quantities of food consumed (in g) or portion size by the nutrient contents per 100 g of food listed in the Chinese food composition tables [23].

### 2.3. Sample Collection and Preparation

The fecal, urine and fingernail samples were collected from the participants in January, April, July, and October. Fresh fecal samples and morning urine samples were maintained at 4 °C for ≤5 h before processing, and then stored at −80 °C until analysis. Fingernail samples were stored in clean separate bags.

### 2.4. Analysis of Metabolites in Feces

SCFAs including acetic acid, propionic acid, butyric acid, isobutyric acid, valeric acid and isovaleric acid in feces were analyzed as described previously [24]. After thawing at room temperature, samples were diluted, vortexed and then centrifuged. Supernatant fractions were then acidified with orthophosphoric acid. Individual SCFAs were separated and quantified by capillary GC (GC-2010 Plus, Shimadzu, Kyoto, Japan). The sum of the concentrations of acetic acid, propionic acid, butyric acid, isobutyric acid, valeric acid and isovaleric acid was calculated as the total SCFA concentration.

The contents of total bile acids in feces were measured by the enzymatic cycling method using a commercial kit (Total Bile Acids Kit; Mindray Bio-Medical Corp., Shenzhen, China) following the manufacturer’s instructions.

Ammonia contents in feces were determined using the indophenol blue procedure as described previously [25]. Fecal specimens were diluted to the appropriate concentration and centrifuged at 13,000× *g* for 20 min. The supernatant fractions were reacted with aqueous phenol plus sodium nitroprusside solution and alkaline sodium hypochlorite. The mixtures were heated for 15 min at 60 °C in a shaking water-bath. After cooling quickly to room temperature, the optical density (625 nm) of the endproduct (indophenol) formed was measured by colorimetry.

### 2.5. Analysis of Metabolites in Urine

Phenol and *p*-cresol in urine were analyzed as described previously [26]. After thawing at room temperature, the samples were centrifuged (9000× *g*, 20 min, at 4 °C). Supernatant fractions were acidified with hydrochloric acid, and then vortexed and heated for 60 min at 90 °C in a water-bath. After cooling quickly to room temperature, ethyl acetate was added for extraction. Phenol and *p*-cresol in the upper ethyl acetate phase were quantified by capillary GC.

The contents of uric acid in urine were analyzed by the uricase-peroxidase colorimetric method using a commercial kit (Uric Acid Kit; Mindray Bio-Medical Corp.) following the manufacturer’s instructions. Urea contents in urine were assayed by the urease-glutamic dehydrogenase colorimetric method using a commercial kit (Urea Kit; Mindray Bio-Medical Corp.) following the manufacturer’s instructions. Creatinine contents in urine were analyzed by the Jaffe colorimetric method using a commercial kit (Creatinine Kit; Mindray Bio-Medical Corp.) following the manufacturer’s instructions.

Ammonia contents in urine samples were also analyzed similarly using the abovementioned indophenol blue procedure.

### 2.6. Analysis of Element Profiles in Nails

The concentrations of elements including Na, Mg, K, Ca, Mn, Fe, Cu, Zn, As, Sn, Sb, Pb, Cr, Co, Ni, Se, Sr, and Ba in fingernails were determined by inductively coupled plasma mass spectrometry (ICP-MS) as described previously [27]. Nitric acid (65%) and hydrogen peroxide (30%) were added to fingernail samples, and then the microwave digestion was carried out. The element concentrations were determined by CRC-ICP-MS (Agilent 7700e ICP-MS with a collision/reaction cell) under optimized conditions.

### 2.7. Statistical Analysis

The results were presented as mean values with their standard deviation. The nonparametric *Kruskal-Wallis* test in SPSS 19.0 software (International Business Machines Corp., Armonk, NY, USA) was performed to analyze the statistical differences among the three groups. In addition, the SCFAs, total bile acids, phenol, *p*-cresol, uric acid, urea, creatinine, ammonia, and element contents were summarized by multivariate statistical analysis. PLS-DA was used to build the classification model among the three groups. PLS-DA was performed with the SIMCA-P 11.5 software package (Umetrics, Umea, Sweden). To minimize the influence of the heteroscedasticity resulting from the concentration variations of different components on the multivariate statistical model, the data set was mean-centered and scaled to unit variance prior to PLS-DA [28,29]. The values of the variable importance in the projection (VIP) obtained from the PLS-DA model were used to identify the differential components contributing to the variations among the three groups. In the VIP plot, each variable is provided with a VIP value and a 95% confidence interval (CI) derived from jack-knifing [30,31,32]. Variables, for which the VIP ± 95% CI exceeds 1, are designated as significant differential components in this analysis [31,32]. Consequently, the characteristic components closely related to centenarians from the LR were determined. For PLS-DA modeling, a typical sevenfold (Leave-1/7th Samples-Out) cross-validation procedure was performed to avoid model over-fitting. The critical *p* value was set as 0.05.

A Spearman correlation test was applied to evaluate correlations between the nutrient intake and metabolites as well as element levels. The statistical significance was set at *p* < 0.05 and *p* < 0.01.

## 3. Results

### 3.1. Nutrient Intake

The nutrient intake levels in the three groups are presented in Table 2. The intake of energy, protein, fat, cholesterol, riboflavin, nicotinic acid and sodium in the LRC group and LRE group were significantly lower than those in the NLRE group (*p* < 0.05). Of these nutrients, energy, protein, fat and cholesterol intake decreased by 20%, 27%, 38% and 54% respectively for the LRC group, and decreased by 19%, 31%, 42% and 48% respectively for the LRE group, compared with those for the NLRE group. However, it is noteworthy that the intake of dietary fiber and vitamin A increased 1.7-fold and 1.4-fold for the LRC group compared with those for the NLRE group (*p* < 0.05). In addition, the intake of vitamin B_6_, folic acid and magnesium in the LRC group was significantly higher than those in the LRE group (*p* < 0.05). Therefore, the results suggest that a lower intake of energy, fat, cholesterol and a higher intake of dietary fiber and vitamin A may have a positive effect on health and longevity. Interestingly, as for most of the nutrients, the intakes in the LRE group were more similar to those in the LRC group than to those in the NLRE group, probably due to the similar dietary habits and dietary pattern of elderly people in the LR. This suggests a major influence of geographical origin on the nutrient intakes of healthy elderly people.

### 3.2. Metabolites in Feces

The contents of acetic acid and total SCFA increased 1.4-fold and 1.3-fold for the LRC group compared with those for the LRE group (*p* < 0.05), and increased 1.8-fold and 1.9-fold for the LRE group compared with those for the NLRE group (*p* < 0.05) (Table 3). In addition, the contents of butyric acid and valeric acid in the LRC group were significantly higher than in the other two groups (*p* < 0.05). Butyric acid concentrations increased 2.1-fold and 3.4-fold for the LRC group, and valeric acid concentrations increased 1.4-fold and 1.8-fold for the LRC group, compared with those for the LRE group and NLRE group, respectively. Besides, the contents of propionic acid, isobutyric acid and isovaleric acid in the LRC group and LRE group were significantly higher than those in the NLRE group (*p* < 0.05). Additionally, the content of total bile acids in the LRC group was significantly higher than in the other two groups (*p* < 0.05). Based on the above mentioned indexes, the results showed that the contents of SCFAs and total bile acids in feces were significantly higher in the LRC group, indicating that the increase of fecal SCFAs and total bile acids has a positive impact on the health and longevity of the centenarians from the LR.

### 3.3. Metabolites in Urine

The contents of phenol, *p*-cresol and urinary ammonia in the LRC group were significantly lower than in the other two groups (*p* < 0.05) (Table 4). Phenol, *p*-cresol and urinary ammonia concentrations decreased by 32%, 33% and 40% respectively for the LRC group compared with those for the LRE group, and decreased by 48%, 40% and 42% respectively for the LRC group compared with those for the NLRE group. Additionally, the contents of uric acid in the LRC group and LRE group were significantly lower than that in the NLRE group (*p* < 0.05). The contents of creatinine in the LRC group and LRE group were significantly higher than that in the NLRE group (*p* < 0.05), and the creatinine contents in the three groups were within the reference range provided in the manufacturer’s instructions. Consequently, the results indicate that lower contents of phenol, *p*-cresol, ammonia and uric acid have a positive influence on the health and longevity of the centenarians from the LR.

### 3.4. Element Levels in Fingernails

The contents of 18 chemical elements in fingernails were determined using ICP-MS, as shown in Table 5. The contents of Mn, Fe, Cu and Co in the LRC group and LRE group were significantly higher than those in the NLRE group (*p* < 0.05). Mn, Fe, Cu and Co contents in the LRC group increased 8.6, 2.4, 1.2 and 4.3-folds respectively, compared with those for the NLRE group (*p* < 0.05). Additionally, Zn content in the LRC group was significantly higher than that in the NLRE group (*p* < 0.05), and the content increased 1.2-fold. Besides, Se content increased 1.8-fold for the LRC group compared with that for the LRE group (*p* < 0.05). In addition, Pb content in the LRC group was the lowest among three groups (*p* < 0.05). Therefore, based on the element profiles in fingernails, the results revealed that higher levels of Mn, Fe, Cu, Co, Zn and Se, as well as a lower level of Pb have a positive influence on the health and longevity of the centenarians from the LR.

### 3.5. Pattern Recognition Analysis

**Figure 2:**
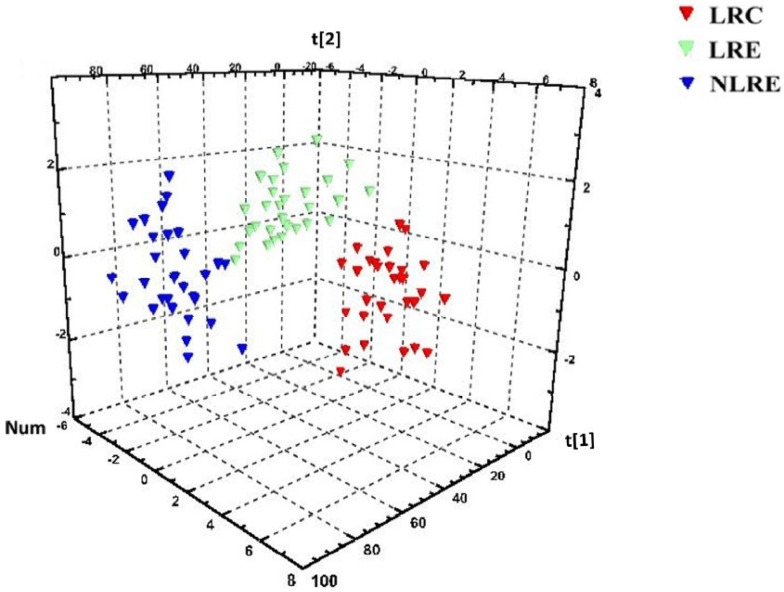
Scores plot from the partial least squares-discriminant analysis of overall indexes including the metabolites and elements in the three groups. Red symbols represent the participants in the LRC group. Green symbols represent the participants in the LRE group. Blue symbols represent the participants in the NLRE group. Their spatial distribution reveals the variations of the metabolites and element profiles among the three groups. The three groups exhibit a clear separation, indicating that there are differences in the metabolites and element profiles among the three groups. LRC, centenarians from longevous region; LRE, elderly people aged 80–99 years from longevous region; NLRE, elderly people aged 80–99 years from a non-longevous region.

In order to comprehensively analyze the overall impact of metabolite and element levels on health and longevity, pattern recognition technology was used to build the classification model and to identify the characteristic components closely related to the centenarians from the LR. The PLS-DA scores plot revealed a clear separation among the three groups, as shown in Figure 2. This indicated that the three groups had distinct differences in the metabolites and element profiles, and the intergroup differences were more significant, though there was some intrinsic physiological variability in each person. Therefore, the results showed that there were distinctive features of metabolites and element profiles in the LRC group.

**Figure 3:**
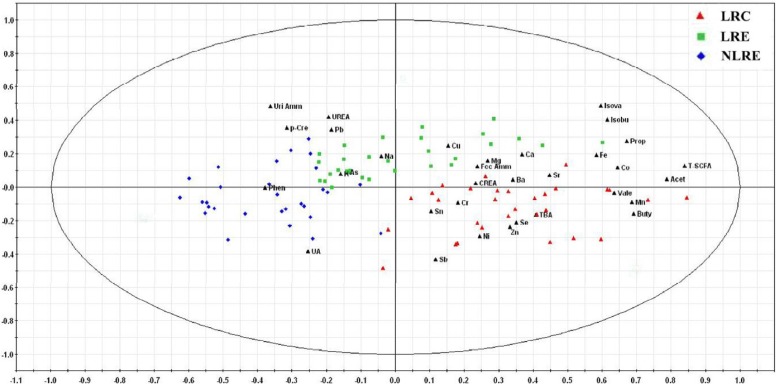
Bi-plot from the partial least squares-discriminant analysis of overall indexes including the metabolites and elements in the three groups. Red triangles represent the participants in the LRC group. Green squares represent the participants in the LRE group. Blue rhombuses represent the participants in the NLRE group. Their spatial distribution reveals the variations of the metabolites and element profiles among the three groups. The three groups exhibit a clear separation, indicating that there are differences in the metabolites and element profiles among the three groups. Moreover, the bi-plot showed that the contents of some beneficial components, such as short chain fatty acids (SCFAs), Mn, Co, Zn and Se, in the LRC group were higher than those in the other two groups. Meanwhile, the contents of some harmful components, such as *p*-cresol, phenol and ammonia, in the NLRE group were higher. LRC, centenarians from longevous region; LRE, elderly people aged 80–99 years from longevous region; NLRE, elderly people aged 80–99 years from a non-longevous region; Acet, acetic acid; Prop, propionic acid; Buty, butyric acid; Isobu, isobutyric acid; Vale, valeric acid; Isova, isovaleric acid; T-SCFA, total SCFA; TBA, total bile acids; Phen, phenol; *p*-Cre, *p*-cresol; UA, uric acid; CREA, creatinine; Fec Amm, fecal ammonia; Uri Amm, urinary ammonia.

In addition, the bi-plot of PLS-DA model showed that the contents of some beneficial components, such as SCFAs, Mn, Co, Zn and Se, in the LRC group were higher than those in the other two groups (Figure 3). Meanwhile, the contents of some harmful components, such as *p*-cresol, phenol and ammonia, in the NLRE group were higher than those in the other two groups.

**Figure 4:**
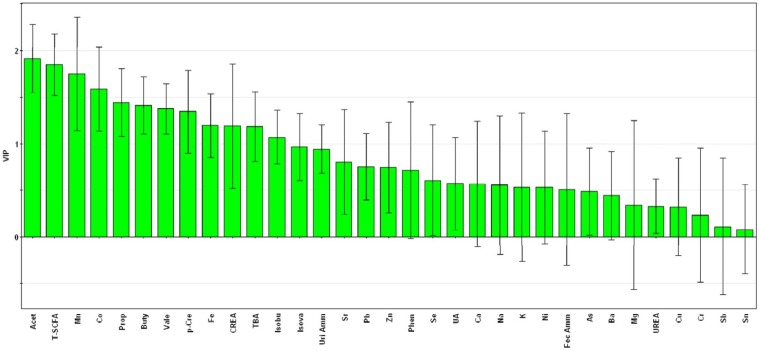
Variable importance in the projection (VIP) plot from the partial least squares-discriminant analysis of overall indexes including the metabolites and elements in the three groups. Variables for which the VIP ± 95% confidence interval (CI) exceeds 1 are designated as significant differential components, including acetic acid, total SCFA, Mn, Co, propionic acid, butyric acid and valeric acid. Acet, acetic acid; Prop, propionic acid; Buty, butyric acid; Isobu, isobutyric acid; Vale, valeric acid; Isova, isovaleric acid; T-SCFA, total SCFA; TBA, total bile acids; Phen, phenol; *p*-Cre, *p*-cresol; UA, uric acid; CREA, creatinine; Fec Amm, fecal ammonia; Uri Amm, urinary ammonia.

To identify which components were accountable for the separation, the VIP plot of the PLS-DA model was used to select the differential components among the three groups (Figure 4). VIP statistics ranked the overall contribution of each component to the PLS-DA model. The VIP analysis identified seven components, including acetic acid (VIP = 1.914), total SCFA (VIP = 1.848), Mn (VIP = 1.753), Co (VIP = 1.587), propionic acid (VIP = 1.447), butyric acid (VIP = 1.418) and valeric acid (VIP = 1.377), as the significant and reliable differential components contributing to the variations among the three groups.

**Figure 5:**
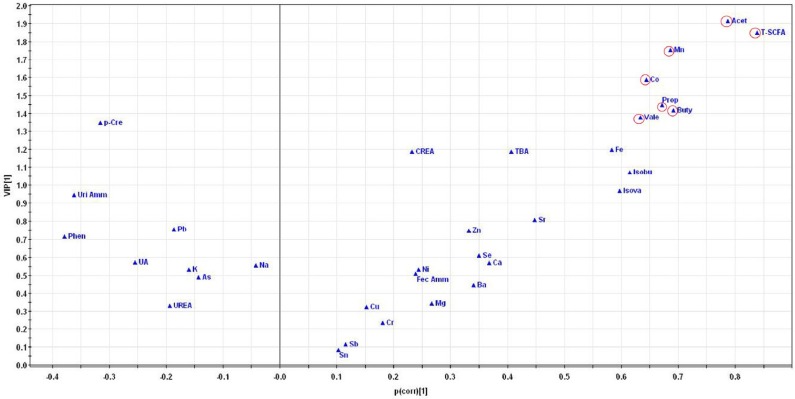
V plot of the partial least squares-discriminant analysis of overall indexes including the metabolites and elements in the three groups. Each triangle denotes an individual component. The triangles far away from the origin represent the components responsible for the differences among the three groups. The acetic acid, total SCFA, Mn, Co, propionic acid, butyric acid and valeric acid are the farthest away from the origin in the V plot, which further indicates that they are the differential components obtained from the PLS-DA model. Acet, acetic acid; Prop, propionic acid; Buty, butyric acid; Isobu, isobutyric acid; Vale, valeric acid; Isova, isovaleric acid; T-SCFA, total SCFA; TBA, total bile acids; Phen, phenol; *p*-Cre, *p*-cresol; UA, uric acid; CREA, creatinine; Fec Amm, fecal ammonia; Uri Amm, urinary ammonia.

Besides, the differential components were further verified by the V plot of the PLS-DA model, as shown in Figure 5. In the V plot, each triangle denoted an individual component. The triangles far away from the origin represented the components responsible for the differences among the three groups. The abovementioned differential components, including acetic acid, total SCFA, Mn, Co, propionic acid, butyric acid and valeric acid, were the farthest away from the origin in the V plot, which further indicated that they were the differential components obtained from the PLS-DA model.

The univariate statistical analysis (*Kruskal-Wallis* test) was used to validate the statistically significant difference of the differential components derived from multivariate statistical analysis. There were significant differences in acetic acid (*p* = 0.000), total SCFA (*p* = 0.000), Mn (*p* = 0.000), Co (*p* = 0.000), propionic acid (*p* = 0.000), butyric acid (*p* = 0.000) and valeric acid (*p* = 0.000) among the three groups; the concentrations of these differential components in the LRC group were relatively higher. Therefore, the seven significant differential components could serve as the characteristic components closely related to centenarians from the LR.

### 3.6. Correlation Analysis

**Figure 6:**
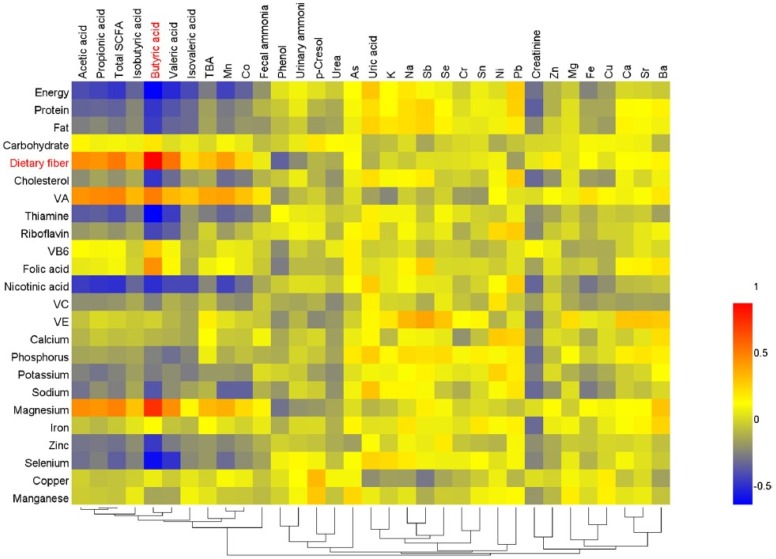
Correlations between the nutrient intake and metabolites as well as element levels. Red denotes positive correlation, blue denotes negative correlation, and yellow denotes no correlation. There was a significant positive correlation between dietary fiber intake and butyric acid contents in feces, and the correlation coefficient was highest (*r* = 0.896, *p* < 0.01). The other short chain fatty acids also were positively associated with dietary fiber intake (*p* < 0.05). Besides, the dietary fiber intake was negatively associated with the contents of phenol in urine (*r* = −0.326, *p* < 0.01).

In order to assess the relationships between the nutrient intake and metabolites, as well as element levels, the Spearman correlation test was performed (Figure 6). There was a significant positive correlation between dietary fiber intake and butyric acid contents in feces, and the correlation coefficient was highest (*r* = 0.896, *p* < 0.01). The other short chain fatty acids were also positively associated with dietary fiber intake (*p* < 0.05). In addition, the contents of the SCFAs were also significantly associated with the intake of energy, protein, vitamin A and nicotinic acid (*p* < 0.05). Besides, the contents of phenol were negatively associated with the intake of dietary fiber (*r* = −0.326, *p* < 0.01). Additionally, Mn contents in fingernails were significantly associated with the intake of energy, protein, fat, dietary fiber, cholesterol, vitamin A, thiamine, nicotinic acid, sodium, magnesium, zinc and selenium (*p* < 0.05). Co contents in fingernails were significantly associated with the intake of energy, dietary fiber, cholesterol, vitamin A, thiamine, nicotinic acid, sodium and magnesium (*p* < 0.05).

## 4. Discussion

In this study, we captured the characteristics of metabolites in feces and urine and element profiles in the fingernails of healthy centenarians from the LR using pattern recognition analysis combined with a univariate statistical test. As a result, the LRC group showed a distinct metabolic pattern. Seven characteristic components closely related to the centenarians from the LR were identified. The concentrations of the characteristic components, including acetic acid, total SCFA, Mn, Co, propionic acid, butyric acid and valeric acid, were significantly higher in the LRC group (*p* < 0.05). Additionally, the further correlation analysis revealed that there were some significant correlations between nutrient intake and metabolites. Therefore, these results confirm the original research hypothesis that there would be distinctive features of metabolites and element profiles in healthy centenarians from the LR, and that these would be associated with nutrient intake.

SCFAs in the gut perform various physiological functions [33,34], which are recognized as essential energy sources and act as signal transduction molecules via G-protein coupled receptors [35]. Butyric acid and propionic acid can activate intestinal gluconeogenesis, which promote metabolic benefits via gut-brain neural circuits [36]. Besides, SCFAs have anti-inflammatory, antitumorigenic, and antimicrobial effects [33], and are also associated with a lower risk for some diseases, such as irritable bowel syndrome, inflammatory bowel diseases, cardiovascular diseases and cancer [33,37]. By contrast, phenol, *p*-cresol and ammonia are potential cytotoxins and carcinogens, and are related to a greater risk of cancers [38]. However, the relevant reports on the metabolites including SCFAs, phenol, *p*-cresol, and ammonia of elderly people are rather limited, especially for centenarians. As for this study, we discovered that the levels of acetic acid, propionic acid, butyric acid, valeric acid and total SCFA in the LRC group were significantly higher (*p* < 0.05), which were identified as characteristic components closely related to the centenarians, whereas the contents of phenol, *p*-cresol and urinary ammonia in the LRC group were significantly lower than in the other two groups (*p* < 0.05). Together, the results indicate that for elderly people, the relatively higher levels of SCFAs and lower levels of phenol, *p*-cresol and ammonia are conducive to the longevity of the centenarians from the LR. More interestingly, as for most of the SCFAs in feces, the contents in the LRE group were more similar to those in the LRC group than to those in the NLRE group, which could be attributed, firstly, to the similar dietary habits, dietary pattern and lifestyle of elderly people in the LR; secondly, to the same geographical environment in the LR. This suggests an important influence of geographical origin on the metabolites of healthy elderly people. It has been reported that diet, lifestyle and other environmental factors impact on the metabolite levels [39,40] and give rise to regional metabolomic phenotypes [41].

As for the elements in fingernails, Mn is an essential cofactor of mitochondrial superoxide dismutase (SOD) which is a key antioxidant enzyme [42,43]. Moreover, Mn is known as an important anti-aging element [19]. Co plays a biologically essential role as a cofactor in a number of proteins, and also a component of the vitamin B_12_ complex [44]. In this study, we discovered that the contents of Mn and Co in the LRC groups were significantly higher (*p* < 0.05), which were identified as characteristic components closely related to the centenarians. Therefore, the results suggested that the two characteristic elements also provided an essential material guarantee for health and longevity of the centenarians from the LR.

More importantly, we comprehensively analyzed the overall effect of metabolites and elements using the PLS-DA method. This pattern recognition method can distinguish the ‘among-groups’ and ‘within-groups’ variability, and thereby extract the most relevant information in differences among groups to build the classification model [14,45]. Therefore, the PLS-DA model can maximize systematic variations among groups, and thus achieve global separation profile among groups [46]. Additionally, PLS-DA plays a unique role in the identification of the most important variables and handling of highly collinear data [47,48]. Moreover, the method has distinct advantages in constructing the classification model for a small number of samples, which can overcome the limitation of a small sample size [49,50,51]. Relevant studies have shown that PLS-DA is much more robust for a small sample size [45]. For example, the *Irs1*^−/−^ model group (*n* = 5) and WT control group (*n* = 5) were clearly distinguished [51]. Therefore, based on the advantages of PLS-DA, the method was used for pattern recognition in the present study and the results of the analysis are reliable and efficient. PLS-DA results revealed a unique metabolic pattern of the centenarians from the LR, and seven characteristic components were identified that are closely related to the centenarians. In consideration of the important physiological functions of these characteristic components, the specific metabolic pattern of the LRC group should have an important and positive influence on health and longevity. Additionally, the results suggested that PLS-DA could be used to manifest the different metabolic patterns of different populations. In a previous study on urine metabolic profiling of northern Italian centenarians, Collino et al. found that compared with the elderly group, the contents of phenylacetylglutamine, *p*-cresol-sulfate, and 2-hydroxybenzoate in centenarians were significantly higher [13]. There are some differences from our results, indicating that the centenarians from Bama LR have a unique metabolic pattern. However, there are very few researches on using PLS-DA to depict the comprehensive feature of metabolites and elements in healthy centenarians.

More remarkably, we discovered that dietary fiber intake was positively associated with SCFAs (*p* < 0.05), especially butyric acid (*p* < 0.01), and negatively associated with phenol concentrations (*p* < 0.01). It is therefore concluded that an appropriate increase of dietary fibers in a daily diet is a path toward the formation of the longevity phenomenon. Nevertheless, to date, we have not yet found published data on associating diet with metabolites as well as elements in the fingernails of healthy elderly people over 80 years of age. A previous study reported that SCFAs are produced during bacterial fermentation of undigested carbohydrates, such as non-starch polysaccharides, dietary fibers and resistant starch [33]. β-Glucan, fructo-oligosaccharide, and a mixture of dietary fibers can be converted to butyric acid in vivo [52]. Besides, a study showed that the high-fat diet reduced formation of butyric acid in rats, and the dietary fiber counteracted the harmful effect and stimulated the butyric acid formation [53]. To date, most of the relevant studies focus on investigating the relationships between a specific diet and metabolites by means of dietary intervention [53,54,55]. However, the study on the relations between metabolites and nutrient intakes of healthy elderly people maintaining their usual eating patterns is rather limited. McOrist et al. discovered that the total SCFA and butyric acid concentrations in feces from eight healthy volunteers (age: 31–59 years, *n* = 8) were weakly associated with dietary fat, sugar and carbohydrate intake that were obtained by self-report [56]. There are some differences from our results, which reveals distinct characteristics of metabolites and nutrient intake of the healthy people over 80 years of age. In addition, the differences of the dietary assessment method also could lead to the differences in results. Among available nutrition assessment approaches, the WDR method is the most precise and is accepted as the gold standard [57], though it is time-consuming, expensive, and generally requires considerable commitment on the part of the volunteers [23]. In this study, four season consecutive 7-day WDRs were carried out to assess the habitual intake of nutrients, which represents real life intake and minimizes variances in dietary intake according to seasons and days.

The present study has several strengths, including the use of the four season consecutive 7-day WDRs method, which provides a more accurate and robust report of elderly people’s usual intake. Furthermore, we pooled data of metabolites and elements obtained in January, April, July, and October to minimize intra-individual variation according to seasons and days. Additionally, the three kinds of different biological samples, including feces, urine, and fingernails, were analyzed to comprehensively assess the features of metabolites and element profiles in healthy centenarians from different perspective. Lastly, seven characteristic components closely related to centenarians from the LR were discovered using the PLS-DA method which is robust and reliable for a small sample size.

However, some limitations of this preliminary exploratory study need to be noted. First, health and longevity also depend on many other factors, such as genes and environment factors [58], while our study is limited by its cross-sectional design, therefore a causal relation between metabolites or elements and longevity cannot be concluded. Nevertheless, this study discovered the unique metabolic pattern of the centenarians from the LR, which provided new clues for further research on the relationship between longevity and metabolism. Future studies are needed to explore the mechanisms of metabolites or elements impacting healthy aging and the important metabolic pathways affecting aging. Second, the detailed data regarding the smoking history and amount were unavailable, and hence it is necessary to exclude the confounding influence of smoking in future studies. Third, the rather limited number of healthy centenarians and elderly people aged 80–99 years, as well as the strict screening criteria, led to a relatively small sample size. Besides, because of the tedious process of the 28-day weighed dietary records method and the complex protocols of sample collection, many participants withdrew from the study. However, we applied the PLS-DA method to perform pattern recognition, which overcomes the limitation of a small sample size. Further studies including populations from other different longevous regions are required to confirm these findings.

## 5. Conclusions

In summary, to our knowledge, this is the only study on the relationships between the nutrient intake and metabolites of healthy elderly people over 80 years of age, and the only study to use the PLS-DA method to assess the overall effect of metabolites and elements. The results indicated that the unique metabolic pattern of the LRC group may have an important and positive influence on the formation of the longevity phenomenon. Elevated dietary fiber intake should be a path toward health and longevity. These findings will provide new clues for future research on nutritional metabonomics and element metabolism in healthy elderly people, which will provide precious information for future human metabolism and longevity studies.

## Figures and Tables

**Table 1:** Participant characteristics ^1^.

Characteristics	LRC Group	LRE Group	NLRE Group
Age (year)	103 ± 3	87 ± 5	88 ± 4
Sex (M/F)	11/19	12/18	13/17
Height (cm)	145.9 ± 10.6	150.8 ± 7.7	157.0 ± 12.0
Weight (kg)	43.1 ± 10.0	45.6 ± 6.5	59.1 ± 8.3
Body mass index (kg/m^2^)	20.0 ± 2.8	20.1 ± 3.1	23.9 ± 1.4

^1^ Values are means ± standard deviation (SD). LRC, centenarians from longevous region; LRE, elderly people aged 80–99 years from longevous region; NLRE, elderly people aged 80–99 years from a non-longevous region; M, male; F, female.

**Table 2:** Nutrient intakes in the LRC group, LRE group and NLRE group ^1^.

Nutrient	LRC Group	LRE Group	NLRE Group	*p*
Energy (Kcal)	1220.30 ± 134.60 ^a^	1237.20 ± 154.45 ^a^	1520.10 ± 215.62 ^b^	0.000
Protein (g)	38.90 ± 7.39 ^a^	36.83 ± 8.61 ^a^	53.48 ± 14.84 ^b^	0.000
Fat (g)	42.24 ± 15.78 ^a^	39.16 ± 13.02 ^a^	67.93 ± 25.00 ^b^	0.000
Carbohydrate (g)	172.56 ± 20.54 ^a^	180.12 ± 24.91 ^a^	167.80 ± 32.05 ^a^	0.343
Dietary fiber (g)	23.48 ± 8.26 ^a^	13.90 ± 6.21 ^b^	13.77 ± 5.86 ^b^	0.000
Cholesterol (mg)	110.73 ± 71.64 ^a^	124.43 ± 121.97 ^a^	238.67 ± 130.94 ^b^	0.001
Vitamin A (μgRE)	1308.37 ± 439.39 ^a^	1181.73 ± 370.05 ^a,b^	956.47 ± 496.79 ^b^	0.001
Thiamine (mg)	0.48 ± 0.16 ^a^	0.57 ± 0.14 ^a,b^	0.60 ± 0.11 ^b^	0.002
Riboflavin (mg)	0.61 ± 0.11 ^a^	0.62 ± 0.17 ^a^	0.84 ± 0.30 ^b^	0.007
Vitamin B_6_ (mg)	0.18 ± 0.08 ^a^	0.13 ± 0.06 ^b^	0.17 ± 0.10 ^a,b^	0.040
Folic acid (μg)	67.36 ± 41.75 ^a^	37.81 ± 30.09 ^b^	57.48 ± 23.88 ^a^	0.002
Nicotinic acid (mg)	7.93 ± 2.30 ^a^	7.65 ± 1.86 ^a^	11.88 ± 3.38 ^b^	0.000
Vitamin C (mg)	61.45 ± 20.02 ^a,b^	51.64 ± 19.06 ^a^	68.49 ± 23.20 ^b^	0.014
Vitamin E (mg)	8.16 ± 3.65 ^a,b^	6.28 ± 3.41 ^a^	9.15 ± 3.95 ^b^	0.014
Calcium (mg)	481.90 ± 87.48 ^a,b^	421.93 ± 127.94 ^a^	511.83 ± 158.33 ^b^	0.003
Phosphorus (mg)	602.77 ± 74.36 ^a,b^	569.13 ± 113.71 ^a^	779.93 ± 223.26 ^b^	0.001
Potassium (mg)	1433.00 ± 203.42 ^a,b^	1269.13 ± 197.30 ^a^	1546.33 ± 252.80 ^b^	0.000
Sodium (mg)	1817.67 ± 222.17 ^a^	1854.26 ± 474.14 ^a^	2144.78 ± 471.59 ^b^	0.000
Magnesium (mg)	354.73 ± 71.19 ^a^	283.73 ± 78.90 ^b^	276.95 ± 80.48 ^b^	0.000
Iron (mg)	14.64 ± 4.40 ^a^	13.85 ± 3.69 ^a^	15.26 ± 3.11 ^a^	0.058
Zinc (mg)	5.40 ± 1.35 ^a^	6.10 ± 1.74 ^a^	6.35 ± 1.96 ^a^	0.062
Selenium (μg)	13.86 ± 5.36 ^a^	15.36 ± 4.82 ^a,b^	17.68 ± 4.10 ^b^	0.001
Copper (mg)	2.08 ± 2.11 ^a^	3.09 ± 2.12 ^a^	2.06 ± 1.89 ^a^	0.067
Manganese (mg)	3.19 ± 0.83 ^a^	3.60 ± 0.70 ^a^	3.68 ± 1.16 ^a^	0.145

^1^ Values are means ± SD. Values without a common superscript letter in a row were significantly different, *p* < 0.05. The *p* values are obtained by *Kruskal-Wallis* test. LRC, centenarians from longevous region; LRE, elderly people aged 80–99 years from longevous region; NLRE, elderly people aged 80–99 years from a non-longevous region.

**Table 3:** Metabolites in feces in the LRC group, LRE group and NLRE group ^1^.

Metabolite	LRC Group	LRE Group	NLRE Group	*p*
Acetic acid (µg/g)	2539.47 ± 875.80 ^a^	1825.13 ± 527.79 ^b^	1016.17 ± 644.02 ^c^	0.000
Propionic acid (µg/g)	875.53 ± 363.69 ^a^	830.80 ± 506.01 ^a^	326.67 ± 214.75 ^b^	0.000
Isobutyric acid (µg/g)	195.03 ± 75.92 ^a^	186.18 ± 113.04 ^a^	109.51 ± 67.54 ^b^	0.000
Butyric acid (µg/g)	780.61 ± 587.01 ^a^	365.33 ± 291.05 ^b^	226.99 ± 153.17 ^b^	0.000
Isovaleric acid (µg/g)	358.19 ± 184.83 ^a^	388.27 ± 254.08 ^a^	185.15 ± 138.60 ^b^	0.000
Valeric acid (µg/g)	223.48 ± 76.80 ^a^	157.93 ± 93.42 ^b^	121.77 ± 49.31 ^b^	0.000
Total SCFA (µg/g)	4972.31 ± 1773.99 ^a^	3753.63 ± 1355.86 ^b^	1986.27 ± 1175.16 ^c^	0.000
Total bile acids (μmol/g)	0.15 ± 0.06 ^a^	0.10 ± 0.04 ^b^	0.08 ± 0.07 ^b^	0.000
Fecal ammonia (mg/g)	0.68 ± 0.28 ^a^	0.59 ± 0.22 ^a^	0.56 ± 0.33 ^a^	0.126

^1^ Values are means ± SD. Values without a common superscript letter in a row were significantly different, *p* < 0.05. The *p* values are obtained by *Kruskal-Wallis* test. LRC, centenarians from longevous region; LRE, elderly people aged 80–99 years from longevous region; NLRE, elderly people aged 80–99 years from a non-longevous region.

**Table 4:** Metabolites in urine in the LRC group, LRE group and NLRE group ^1^.

Metabolite	LRC Group	LRE Group	NLRE Group	*p*
Phenol (mg/L)	13.32 ± 16.02 ^a^	19.60 ± 8.26 ^b^	25.79 ± 29.46 ^b^	0.003
*p*-Cresol (mg/L)	65.52 ± 25.08 ^a^	98.26 ± 31.50 ^b^	109.52 ± 40.77 ^b^	0.000
Uric acid (μmol/L)	974.63 ± 525.26 ^a^	830.97 ± 251.23 ^a^	1147.03 ± 191.99 ^b^	0.000
Urea (mmol/L)	285.02 ± 132.62 ^a^	371.80 ± 106.73 ^b^	325.96 ± 151.43 ^ab^	0.027
Creatinine (μmol/L)	7672.50 ± 5840.73 ^a^	5776.94 ± 1258.51 ^a^	3791.72 ± 1119.87 ^b^	0.000
Urinary ammonia (μg/μL)	0.29 ± 0.15 ^a^	0.48 ± 0.17 ^b^	0.50 ± 0.35 ^b^	0.001

^1^ Values are means ± SD. Values without a common superscript letter in a row were significantly different, *p* < 0.05. The *p* values are obtained by *Kruskal-Wallis* test. LRC, centenarians from longevous region; LRE, elderly people aged 80–99 years from longevous region; NLRE, elderly people aged 80–99 years from a non-longevous region.

**Table 5:** Element concentrations in fingernails in the LRC group, LRE group and NLRE group ^1^.

Element	LRC Group	LRE Group	NLRE Group	*p*
Na (μg/L)	107.836 ± 106.206 ^a^	136.393 ± 122.729 ^a^	164.905 ± 127.408 ^a^	0.208
Mg (μg/L)	61.143 ± 30.351 ^a^	62.514 ± 18.946 ^a^	53.848 ± 26.503 ^a^	0.332
K (μg/L)	5.658 ± 8.982 ^a^	4.759 ± 7.180 ^a^	18.521 ± 48.124 ^a^	0.355
Ca (μg/L)	869.077 ± 452.755 ^a^	850.038 ± 488.529 ^a^	650.867 ± 407.325 ^a^	0.170
Mn (μg/L)	3.051 ± 2.294 ^a^	1.499 ± 0.822 ^a^	0.353 ± 0.368 ^b^	0.000
Fe (μg/L)	38.153 ± 15.967 ^a^	40.478 ± 25.144 ^a^	16.162 ± 15.351 ^b^	0.000
Cu (μg/L)	4.579 ± 1.409 ^a^	5.547 ± 2.592 ^a^	3.936 ± 3.153 ^b^	0.000
Zn (μg/L)	137.406 ± 43.331 ^a^	118.979 ± 24.022 ^a,b^	112.877 ± 40.170 ^b^	0.012
As (μg/L)	0.138 ± 0.091 ^a^	0.241 ± 0.315 ^a^	0.289 ± 0.521 ^a^	0.061
Sn (μg/L)	1.029 ± 2.994 ^a^	0.567 ± 1.172 ^a^	0.866 ± 1.679 ^a^	0.730
Sb (μg/L)	0.126 ± 0.167 ^a^	0.059 ± 0.071 ^a^	0.111 ± 0.131 ^a^	0.306
Pb (μg/L)	0.140 ± 0.153 ^a^	0.311 ± 0.244 ^b^	0.279 ± 0.181 ^b^	0.000
Cr (μg/L)	0.957 ± 1.058 ^a^	0.763 ± 0.824 ^a^	0.743 ± 1.268 ^a^	0.071
Co (μg/L)	0.030 ± 0.015 ^a^	0.024 ± 0.017 ^a^	0.007 ± 0.010 ^b^	0.000
Ni (μg/L)	2.130 ± 4.765 ^a^	0.410 ± 0.301 ^b^	0.785 ± 0.689 ^a,b^	0.009
Se (μg/L)	0.476 ± 0.293 ^a^	0.267 ± 0.255 ^b^	0.315 ± 0.314 ^a,b^	0.020
Sr (μg/L)	0.561 ± 0.391 ^a^	0.449 ± 0.325 ^a^	0.329 ± 0.246 ^a^	0.058
Ba (μg/L)	0.879 ± 0.783 ^a^	0.745 ± 0.603 ^a^	0.636 ± 0.473 ^a^	0.605

^1^ Values are means ± SD. Values without a common superscript letter in a row were significantly different, *p* < 0.05. The *p* values are obtained by *Kruskal-Wallis* test. LRC, centenarians from longevous region; LRE, elderly people aged 80–99 years from longevous region; NLRE, elderly people aged 80–99 years from a non-longevous region.
